# Commentary: Surgical reconstruction with the help of a 3-dimensional printer

**DOI:** 10.1016/j.xjtc.2021.09.026

**Published:** 2021-09-16

**Authors:** Charles Laurin, Siamak Mohammadi

**Affiliations:** Department of Cardiac Surgery, Quebec Heart and Lung Institute, Quebec City, Quebec, Canada

**Figure:**
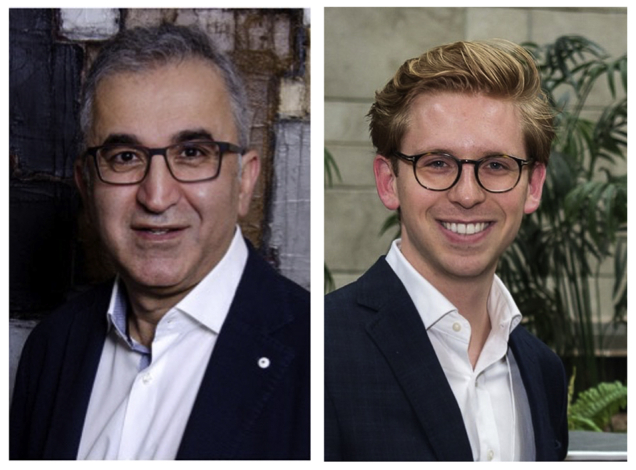
Siamak Mohammadi, MD, FRCSC, and Charles Laurin, MD

**Central Message**

A bioresorbable 3D-printed external splint could be a viable solution for rare, lethal airway agenesis.

See Article page 563.

The evolution of cardiothoracic surgery was made possible by of the actions of courageous surgeons who were keen to tackle complex challenges. In this issue of *JTCVS Techniques*, Tsai and colleagues^1^ report the successful and complete surgical treatment of a Floyd type I tracheal agenesis associated with a congenital valvular heart defect. It is the second report of esophagotracheoplasty using 3-dimensional (3D)-printed external support.^2^

The authors managed this rare case of tracheal reconstruction with a bioresorbable 3D-printed splint to substitute the trachea and to externally support their esophagotracheoplasty. The authors were later able to perform a right colon interposition to restore the gastrointestinal continuity. At more than 36 months of life, the child is alive, has resumed oral nutrition, and has achieved the motor development milestones of a 1-year-old.

The novelty of this approach goes beyond the interests of a handful of specialized pediatric and thoracic surgeons. These customized prostheses are particularly enticing for the younger patient population, where ongoing growth is a major drawback to any permanent reconstruction.^3^ Bioresorbable implants also have the potential advantages to decrease the amount of postoperative fibrosis and erosion into surrounding tissues, lowering the risk of future surgery.

Tsai and colleagues^1^ are to be praised for sharing their strategy for the complex treatment of tracheal agenesis with a bioresorbable splint, with a clear description of surgical steps. Despite the rarity of this congenital condition, the technology could be applied to other pediatric and adult thoracic pathologies requiring temporary internal or external splinting.
